# The Effects of a Multisector, Multilevel Intervention on Child Dietary Intake: California Childhood Obesity Research Demonstration Study

**DOI:** 10.3390/nu15204449

**Published:** 2023-10-20

**Authors:** Shih-Fan Lin, Michelle Murphy Zive, Emily Schmied, Jonathan Helm, Guadalupe X. Ayala

**Affiliations:** 1 Institute for Behavioral and Community Health (IBACH), San Diego, CA 92123, USA; eschmied@sdsu.edu (E.S.); ayala@sdsu.edu (G.X.A.); 2 Division of Health Promotion and Behavioral Science, School of Public Health, San Diego State University, San Diego, CA 92182, USA; 3 Herbert Wertheim School of Public Health, University of California, San Diego, CA 92093, USA; mzive@ucsd.edu; 4 Department of Psychology, San Diego State University, San Diego, CA 92182, USA; jhelm@sdsu.edu

**Keywords:** childhood obesity, healthy diet, caregiver–child intake, parenting strategies, family meal context

## Abstract

Consuming too few fruits and vegetables and excess fat can increase the risk of childhood obesity. Interventions which target mediators such as caregivers’ dietary intake, parenting strategies, and the family meal context can improve children’s diets. A quasi-experimental, pre–post intervention with four conditions (healthcare (HC-only), public health (PH-only), HC + PH, and control) was implemented to assess the effects of the interventions and the effects of the mediators. HC (implemented with the Obesity Care Model) and PH interventions entailed capacity building; policy, system, and environment changes; and a small-scale media campaign to promote healthy eating. Linear mixed models were used to assess intervention effects and the mediation analysis was performed. Predominantly Hispanic/Latino children and caregivers from rural communities in Imperial County, California, were measured at baseline (N = 1186 children/848 caregivers) and 12 months post-baseline (N = 985/706, respectively). Children who were overweight/obese in the HC-only condition (M = 1.32) consumed more cups of fruits at the 12-month follow-up than those in the control condition (M = 1.09; *p* = 0.04). No significant mediation was observed. Children in the PH-only condition consumed a significantly higher percentage of energy from fat (M = 36.01) at the follow-up than those in the control condition (M = 34.94, *p* < 0.01). An obesity intervention delivered through healthcare settings slightly improved fruit intake among at-risk children, but the mechanisms of effect remain unclear.

## 1. Introduction

A risk factor for childhood obesity in the United States (U.S.) is poor diet quality, which includes low intakes of fruits and vegetables (FVs) and excess consumption of fat [1]. About 75% and 91% of children and adolescents aged 2–19 consumed FV, respectively, on a given day in 2015–2018 [2]. Fruit intake was found to decline with age but found to increase with family income; however, no such differences were observed for vegetable intake [2]. In 2017–2018, the percentage of energy from total fat among U.S. youth (2–19 years) was 35.6% [3]. School-based interventions to prevent and control childhood obesity have commonly focused on increasing FVs intake [4] and reducing fat intake among children [5]. These interventions often promote consumption of FVs as a lower fat alternative to savory snacks, for example.

Ecological models have demonstrated that behavior change interventions should target multiple levels of influence [6]. This may be particularly true for interventions targeting dietary intake among children. Research has demonstrated the importance and efficacy of family-based interventions involving parents/caregivers [7]. Additionally, given the importance of social (e.g., diet-related school wellness policies) and physical (e.g., access to water fountains) environmental characteristics on children’s behaviors [8], childhood obesity interventions involving both parents/caregivers and children have been implemented outside the home, including in healthcare facilities [9,10], schools [11], early care and education (ECE) centers [12], and communities [13]. Although some of these interventions were effective in changing children’s diets [9,12] and some were not [10,11,13], research that examines the mechanisms of effect between parent/caregiver dietary behaviors and children’s dietary behaviors is needed.

One potential mechanism through which family-based interventions may impact a child’s diet is through the caregiver’s diet. However, the evidence for this relationship is mixed. Observational research has shown weak [14]–modest [15] associations and/or varying associations between mothers versus fathers and their children. Intervention studies have found improvements in FVs intake among both caregivers and children [5,16] and fat intake among caregivers only [17]. One home-based intervention found that increases in caregiver FVs servings significantly predicted increases in child FVs intake [16]. Another potential mechanism is diet-related parenting strategies (e.g., controlling, monitoring, reinforcement) [18]. For example, increases in child FVs intake were mediated by improvements in parental monitoring and reinforcement and reductions in the use of controlling parenting strategies [19].

Family meal context, such as eating food away from home or having family meals together, are other diet-related parenting practices [20,21,22]. Food away from home (i.e., foods prepared outside of the home) has lower nutritional quality and contains more calories compared to food prepared at home [20]. A review showed that repeated intake of food away from home over time has a significant impact on energy imbalance and weight gain [21]. Family meals together are associated with positive dietary behaviors: a meta-analysis concluded that the association between frequent family meals and better nutritional health outcomes among children is robust [22].

The purpose of this study was to examine the effects of the California Childhood Obesity Research Demonstration Study (CA-CORD) on child diets. CA-CORD was a multisector, multilevel intervention to prevent and control childhood obesity [23]. As one of three studies funded by the Centers for Disease Control and Prevention in 2011, CA-CORD modified policies, systems, and environments to promote four health behaviors (healthy eating, physical activity, water intake, and quality of sleep) in four settings (healthcare facilities, ECE centers, schools, and community). The present study assessed the 12 months post-baseline intervention effects on child diets, specifically cups of fruit, cups of vegetables, and percent energy from fat (%Efat). Although this study was conducted prior to the release of the Dietary Guidelines for Americans 2015–2020 (8th Edition), in which the recommendation of a 35% limit for total dietary fat was removed, total fat reduction remained one of the main targets of dietary intake in this study. The second aim examined the mediating roles of caregivers’ dietary intake, diet-related parenting strategies, and family meal context on the relationship between intervention effects and child diet. Exploratory aims examined the first and second aims in a restricted sample of children who were classified as overweight/obese (BMI percentile ≥85th percentile) based on measured height and weight. The approach of examining intervention effects on the full sample, as well as a restricted sample, helped to determine whether the intervention was effective and for whom.

## 2. Materials and Methods

### 2.1. Study Design

Participants living in three cities (El Centro, Brawley, and Calexico) within Imperial County, California, were assigned to one of four study conditions. The three intervention conditions included combinations of interventions implemented in the healthcare (HC) sector and the public health (PH) sector. A number of interventions were implemented within the HC sector, including expanded provider training of child obesity assessments and community health worker (CHW)-led healthy lifestyle workshops. The HC intervention occurred within a Federally Qualified Health Center (FQHC) with a clinic site in each of the three participating cities. The PH intervention occurred in ECE centers, schools, community recreation organizations, and restaurants. The first condition served as a control with no intervention, the second condition included only HC sector intervention (HC-only), the third condition included only PH sector intervention (PH-only), and the fourth condition included interventions implemented both in the HC and PH sectors and is referred to herein as “HC + PH.” The intervention strategies are described in detail below in Table 1, Section 2.4, and in Ayala et al. [23].

**Figure 1:**
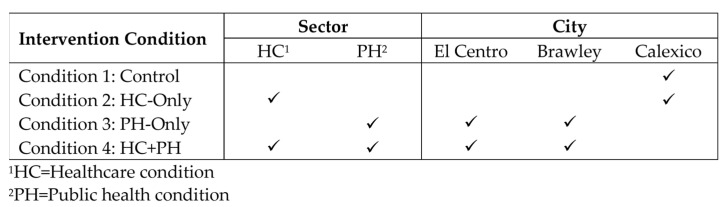
CA-CORD study: condition assignment and sectors involved in each condition.

Participating families were not randomized to condition; rather, condition assignment was based on the families’ location of healthcare source and city of residence. More specifically, PH sector interventions were only implemented in two of the three cities within the region, El Centro and Brawley, whereas the HC sector intervention was implemented within the FQHC clinics in all three cities. Thus, children who were patients of the FQHC and resided in El Centro and Brawley were assigned to the HC + PH condition because it would not be possible to ensure that they were not exposed to any of the intervention activities being implemented within their communities or health clinics. Similarly, children who resided in El Centro and Brawley but who were not patients of the FQHC were assigned to the PH-only condition. Children who resided in Calexico, where the PH sector interventions were not being implemented, and who were patients of the FQHC, were assigned to the HC-only condition. Finally, children residing in Calexico, but who were not patients of the FQHC, were assigned to the control condition. An overview of the study design, including the intervention sector(s) and the participating cities is shown in Figure 1 below.

The study was conducted in Imperial County, CA, located on the U.S.–Mexico border. In 2013, most county residents identified as Hispanic/Latino (81%) and 75% spoke a language other than English at home [24]. Residents also had a disproportionately higher rate of childhood obesity (47%) compared with the state’s residents (38%) in 2010 [25]. The poverty rate in Imperial County (23%) was disproportionately higher than that of California (16%) in 2013 [24].

All study procedures were approved by San Diego State University’s Institutional Review Board. Eligible caregivers provided written informed consent and eligible children over the age of seven provided verbal assent to participate.

### 2.2. Participant Recruitment

A convenience sample of 1186 children and 848 caregivers were enrolled between January 2013 and July 2014. The primary caregiver (e.g., mother, father, grandmother, aunt) and up to two children per household were eligible to participate. The eligibility criteria were: (a) child aged 2–11 years old; (b) child BMI at or above 5th percentile; (c) child not currently taking medication affecting weight other than stimulant medications for attention deficit–hyperactivity disorder; (d) eligible school-age child had to attend one of the participating schools in the CA-CORD study if the child resided in Cities A or B; (e) primary caregiver had to live with the child(ren) for four or more days/week; and (f) family had no plans to move in the subsequent 30 months to allow for an initially longer follow-up period. After eligibility was determined and baseline data were collected, research staff assigned participants to a study condition based on city of residence and whether the child’s usual primary care provider was located at one of the three participating clinic sites in the study. Children’s sex and age as well as caregivers’ preferred language for interview were collected from those who agreed to be screened but did not enroll in the baseline cohort (i.e., non-participants). Due to the study configuration (i.e., the study condition is dependent on the city of residence), it was difficult to anonymize the data for research staff; however, they were unaware of the intervention content and strategies. Finally, participation was discontinued for families moving out of the area during the study period.

### 2.3. Baseline Assessment

The baseline assessment included the following: (a) a caregiver interview with questions related to themselves, the child(ren), and the family (e.g., demographics, child and caregiver sedentary/physical activity/sleep behaviors, parenting strategies); (b) an assessment of the child’s dietary intake in the past week reported by the caregivers and administered by the research staff; and (c) measurements of children’s and caregivers’ height and weight completed by the research staff. Research staff, who were fluent in English and Spanish, completed 24 h of training prior to data collection. Height and weight measurements were completed by 10 trained research staff and the inter-rater reliability on a small convenience subsample (11%) was high (height: r = 0.99; *p* < 0.001; weight: r = 0.99, *p* < 0.001). The same assessment protocol was repeated at the 12-month follow-up. At follow-up, 985 (83.1%) children and 706 (83.3%) caregivers were retained in the study. There was no differential drop-out rate at follow-up by condition (*p* = 0.066).

### 2.4. Intervention Strategies

The CA-CORD intervention strategies were informed by social cognitive theory [26] and family system theory [27]; the study team’s previous research; systematic reviews from the literature; CA-CORD Community Advisory Committee input; and our formative research [23]. Both the HC and PH interventions entailed capacity building as well as policy, system, and environment changes and a small-scale media campaign to promote the four health behaviors. To meet the required sample size, children under five were not required to attend any ECE centers for the PH intervention. In fact, 64.7% of children in the PH-only group either attended a non-participating ECE or did not attend any ECE center—this is slightly lower than the 68.4% in the HC + PH condition. The HC intervention was designed based on the Obesity Care Model (OCM) [28], which considers healthcare delivery and community supports for the prevention and control of childhood obesity. Within the healthcare aspect of the OCM, there are four components: (a) delivery system design to promote quality healthcare for childhood obesity; (b) decision support for healthcare providers utilizing evidence-based guidelines; (c) clinical information systems to promote the use of guideline-concordant care; and (d) self-management support for patients/families. For example, a system-wide change to the electronic health record (EHR) was implemented reminding the pediatrician to generate a four-point treatment plan about the child’s health behaviors with the caregiver during the pediatric visits. To design the four-point treatment plan, child health behavior goals were generated with the caregiver and child, with the latter depending on child age. Goals were printed on the treatment plan and given to the caregiver at the end of the visit. Following the pediatric visits, the patient care coordinator reviewed the child’s four targeted health behaviors with the caregiver and provided sex- and age-specific recommendations for these behaviors. Goals discussed with the pediatrician were refined to maximize achievement of behavioral goals. Goals were designed to be incremental in nature with the patient care coordinator ascertaining where the child was now and realistic next steps to promote the change process (e.g., one additional daily serving of vegetables). Finally, a clinical champion was identified for all three HC intervention sites to support and spearhead intervention implementation.

Families in the HC condition were also invited to participate in a group-based FWP(six workshops) delivered by CHWs. The program was designed to focus on the caregiver–child relationship [29] and the reciprocal influence on each other [30]; details of the program have been described previously [23,31]. Briefly, the CHWs completed 150 h of training on topics such as delivering wellness workshops and conducting motivational interviewing. The workshop topics included techniques for caregiver-modeled health behaviors and effective parenting strategies. Each family also received a total of 12 newsletter (one per month) and 4 motivational interviewing phone calls. Healthy eating intervention strategies are summarized in Table 1, and intervention strategies used to promote other health behaviors are described elsewhere [23]. Examinations of factors affecting implementation of the CA-CORD study across sectors [32] and caregiver engagement in the FWP [31] were reported elsewhere, as were process evaluation results for the ECE sector [33].

### 2.5. Measures

Dependent variables. The three dietary outcomes that were assessed among both children and their caregivers constituted the primary outcome measures: daily cups of fruits, daily cups of vegetables, and %Efat. The collection of dietary intake data was limited given the need to collect a common set of measures across all three sites, and the need to collect other weight-related behavioral data from caregivers and children. Using both child and caregiver dietary intake allows for examining the mediating role of caregiver dietary intake on the relationship between the study condition and the child’s dietary intake.

Child dietary intake was derived from the caregiver report on the English or Spanish version of the 41-item Block Kids Food Screener (BKFS) developed by NutritionQuest; this instrument includes assessment of foods typically consumed in Latin America [34]. BKFS assesses dietary intake of nutrients and food groups among children aged 2–17 years, has good validity against 24 h dietary recall among children aged 10–17 years old; and takes about 10–20 min to complete [34]. To assist with recall of the past seven days, the interviewers were trained to prompt the caregivers to recall the families’ activities during the past week using a hard-copy calendar. Caregivers were encouraged to respond to the best of their knowledge and think about: (a) notable events (e.g., parties, sporting events) to help with recall; (b) what children ate and drank at various locations (e.g., own/family’s/friend’s home, school, snack shops); and (c) everything the child ate or drank including breakfast, lunch, dinner, after school, while watching TV, at bedtime, and on the weekend. Caregivers responded how many days last week the child ate or drank the items on the screener (none last week, 1 day last week, 2 days last week, 3–4 days last week, 5–6 days last week, and every day last week) and how much the child had in one day (e.g., 1, 2, or 3 bowls of cereal; 1, 2, or 3+ glasses of milk). For those who struggled to provide an estimate of usual portion size given variability across days in the past week, the research staff reassured the caregivers to provide their best estimate, reviewing when necessary, and using a day-to-day portion size estimate to calculate an average.

The English version of the BKFS was translated into Spanish by two members of the NutritionQuest team who had experience with a wide variety of dialects. Average daily intake of total calories (kcal); cups of fruits; cups of vegetables, excluding potatoes and legumes; and total grams of fat were computed by NutritionQuest. Total calories from fat were computed by multiplying total grams of fat by 9 (1 g of fat = 9 kcals). Subsequently, child %Efat was computed by taking the percentage of average daily total calories from fat divided by the average daily total calories.

Mediators: Caregiver dietary intake. Caregiver %Efat was estimated using the adult-validated National Cancer Institute Fat Screener. The caregivers were asked how often they consumed each item on the list over the past year (never, less than once/month, 1–3 time(s)/month, 1–2 time(s)/week, 3–4 times/week, 5–6 times/week, 1 time/day, and 2 or more times/day). The reported frequency was first converted into number of times consumed per day (e.g., 1–2 times per week = 0.214 time per day) then multiplied by (a) the corresponding sex- and age-specific median serving size and (b) the sex-specific estimated regression coefficients for each food item. Finally, the %Efat was calculated as the sum of these products across food items [35]. The translation of the fat screener was not validated but it was translated and used in previous randomized controlled trials. Caregivers were asked two separate questions about how many cups of (a) fruits (including 100% pure fruit juice) and (b) vegetables (including 100% pure vegetable juice) they ate or drank each day in the past week (none, ½ cup or less, ½–1 cup, 1–2 cups, 2–3 cups, 3–4 cups, 4 or more cups) [36]. A call-out box illustrating 1-cup equivalents (e.g., 1 cup fruit = 1 small apple) was placed in the survey to help respondents recall portion size. The FVs intake questions were translated by a certified translator followed by an adjudication process, during which all items were reviewed by proficient bilingual investigators and staff to ensure conceptual and linguistic relevance in both languages. The categorical responses were imputed with mid-point (e.g., an estimate of 2–3 cups was imputed as 2.5 cups) to create two separate continuous variables for daily cups of FVs. 

Mediators: Parenting strategies. Parenting strategies were assessed using a validated bilingual scale, the Parenting for Eating and Activity Scale (PEAS) [37]. The original scale was administered to respondents using either an English or Spanish version, depending on the language preference of the caregiver. It consisted of 26 items representing 5 domains associated with child diet- and activity-related behaviors. The PEAS items used in the present study were limited to those pertaining to diet-related parenting strategies, per the scale protocol described in Arredondo et al., [18] which focused on diet intake and physical activity items separately. The scale items assessed monitoring, discipline, and reinforcement (response options range from 1 (never) to 5 (always)), as well as limit setting and control (response options range from 1 (disagree) to 5 (agree)) strategies. Additionally, a factor analysis of the diet-related parenting strategies items in this study sample revealed an additional subscale: “permission seeking” (results available upon request). One item from the monitoring subscale (i.e., I limit the number of snacks my child eats) and another item from the discipline subscale (i.e., I limit the amount of soda my child drinks), described in Arredondo et al. [18], were moved to the new “permission seeking” subscale. A subscale summary score was computed by calculating the means of all items within each subscale. The final six subscales were comprised of 16 items: monitoring (4 items; α = 0.84), discipline (2 items; α = 0.84), reinforcement (1 item), limit setting (2 items; α = 0.63), control (5 items; α = 0.66), and permission seeking (2 items; α = 0.75). Higher scores in each subscale suggested more frequent use or higher agreement with the parenting strategy.

Mediators: Family meal context. The variables to assess family meal context included: frequency of food away from home and family meals together. The caregiver responded to these items for the family; thus, enrolled children (up to two) from the same family received the same response. Two variables assessed food away from home: “During the past week, how many days did your family eat at or bring home food from (a) relatives’ or friends’ home and (b) restaurants?” (including but not limited to fast food, sit-down, or buffet restaurants; takeout and delivery were also considered) [38]. For family meals together, three types of meals were assessed: “During the past week, how many days did you and your child (other family members could be present) eat together for (a) morning meal, (b) afternoon meal/snack, and (c) evening meal?” [39].

Covariates. Both child and caregiver characteristics were included in the models: age, sex, Hispanic/Latino ethnicity (yes/no), foreign-born (yes/no), and language use (English only vs. English and another language or another language only). Additional caregiver/family characteristics such as marital status (married vs. not married), education (less than high school vs. high school or more), employment status (yes/no), home ownership (yes/no), household size (continuous), participation in public food assistance program (e.g., SNAP, WIC; yes/no), and family income to poverty ratio (PIR) were also included. The U.S. Department of Health and Human Services’ 2011 poverty guidelines [40] were used to obtain poverty cutoff values to calculate the PIR. A ratio of 1.0 or greater indicated the income was above the poverty level and a ratio lower than 1.0 indicated the opposite.

BMI Classification. Child BMI percentile was computed using measured height and weight, and sex-specific BMI-for-age growth charts published by CDC [41]. Children who had a percentile at 85% or above were classified as overweight/obese. Caregiver BMI was also computed with measured height and weight using CDC’s formula: BMI = weight (kg)/[height (m)]^2^.

### 2.6. Data Analysis

Outcome analyses. To assess for bias associated with condition assignment and child and caregiver baseline characteristics were compared across conditions using one-way ANOVA for continuous variables and chi-square tests for categorical variables. To assess the intervention effects on daily dietary intake, child fruit intake (cups), vegetable intake (cups), and %Efat were compared at 12-month follow-up while adjusting for baseline values and sociodemographic characteristics that were significantly different across conditions (see covariates section above). Intervention effects were assessed by comparing the three intervention conditions (HC-only; PH-only; HC + PH) against the control condition in linear mixed models to adjust for the family clusters. The mixed modeling assumed outcome data were missing at random. The assumption of normality and common variance were met. The beta coefficients, standard errors, *p*-values, and adjusted means for each condition are reported. Analyses were performed with both the full sample and the restricted sample and the *p*-value was adjusted using the Benjamini–Hochberg method [42] for each family of outcomes due to multiple testing.

Mediation analyses. Mediation analyses were performed using MacKinnon et al.’s protocol [43]. Three sequential regression models were fitted to yield parameter estimates and standard errors. Model 1 examined the intervention effects on all study outcomes as described above. Model 2 examined the intervention effects on all mediators (caregiver dietary intake, parenting strategies, and family meal context) following the same procedures described for outcome analyses. Based on the results of Models 1 and 2, the intervention effects and significant mediators were included in the same model for each significant outcome (Model 3). Given some results from Models 1 and 2 were opposite of the hypothesized direction, these significant outcomes/mediators were not included in the mediation analyses (i.e., Model 3). The mediation analyses were performed on the full and/or restricted sample(s) depending on the results of outcome analyses. The same set of sociodemographic covariates were added to all mediation models. According to MacKinnon et al., the mediated effect is the product (ab) of unstandardized coefficient of the intervention effect (coefficient a) in Model 2 and the unstandardized coefficient of mediators adjusted for intervention effect (coefficient b) in Model 3. The coefficient of ab and 95% confidence interval of ab are reported here. Finally, because only 1.6% (n = 16) of caregivers in the sample were male, sensitivity analyses were conducted by dropping male caregivers. The results were virtually identical, so male caregivers were ultimately retained in the final sample to maximize power. All outcome analyses were performed on Stata/SE 16.1 (Stata version 16.1, Stata Corp., College Station, TX, USA, 2019) and mediation analyses were performed in MPlus (MPlus version 8, Muthén, L.K. and Muthén, B.O., Los Angeles, CA, USA, 2021).

## 3. Results

### 3.1. Non-Participants vs. Participants

Non-participants who were screened but did not enroll in the baseline cohort did not differ from participants in child sex or age. Non-participating caregivers were more likely to report English as their preferred language (*p* < 0.001).

### 3.2. Participants’ Baseline Characteristics

An equal proportion of boys versus girls were recruited across conditions; their mean age was 6.4 years old (see Table 2). Most children (98.5%) were Hispanic/Latino. Children’s average daily fruit and vegetable intake were 1.4 (SD = 0.9) and 0.6 (SD = 0.4) cups, respectively, and %Efat was 35% (SD = 4.6). The mean BMI percentile was 76.3% (SD = 26.5). Several child characteristics were different across conditions as shown in the overall ANOVA/chi-square *p*-values. Based on the descriptive statistics, the children in the PH-only condition were slightly younger (M = 6.0; SD = 2.6; *p* < 0.05); the HC-only condition had the most foreign-born children (13.8%; *p* < 0.01); the PH-only condition had the most children who spoke only English (19.5%; *p* < 0.01).

Caregivers were mostly female (98.4%) and were the mothers of the children (93.2%). Their mean age was 35.7 years old (SD = 8.5). Most caregivers identified as Latino (97.5%) and were married (72.7%). Average daily fruit and vegetable intakes among caregivers were 1.6 (SD = 1.3) and 2.0 (SD = 1.3) cups, respectively, and mean %Efat was 31.2 (SD = 4.2). The average household size was 4.7 (SD = 1.5). Several caregiver/household characteristics were significantly different across the four conditions, as shown in the overall ANOVA/chi-square *p*-values. Based on the descriptives, caregivers in the control condition were slightly older (M = 37.0, SD = 9.1; *p* < 0.05); caregivers in the HC-only condition were most likely to be foreign-born (75.1%; *p* < 0.01) and least likely to only speak English (2.5%; *p* < 0.01); caregivers in the HC + PH condition were least likely to obtain a high school degree and above (59.4%; *p* < 0.01); and caregivers in the control condition were most likely to be employed (45.1%; *p* < 0.01), own a home (30.9%; *p* < 0.01), have higher PIR (M = 1.2, SD = 1.0; *p* < 0.01), have slightly lower BMI (M = 30.3, SD = 6.2; *p* < 0.01), and were the least likely to participate in public food assistance programs (58.0%; *p* < 0.01).

Finally, there were no differences in children’s dietary outcomes between those who completed the 12-month follow-up assessment versus those who did not complete the follow-up assessment. However, families that completed the follow-up assessment were more culturally traditional (e.g., children less likely to speak English only; *p* < 0.01) and more likely to participate in a public food assistance program (*p* = 0.03).

### 3.3. Intervention Effects on Child Dietary Outcomes and Potential Mediators

Child dietary outcomes. Table 3 shows the intervention effects on child diet. For child daily fruit intake in the full sample, no significant intervention effect was found at follow-up. In the restricted sample, a significant intervention effect showed that children in the HC-only condition consumed significantly more cups of fruit than children in the control condition (M = 1.32 vs. M = 1.09, *p* = 0.04). For child vegetable intake, no significant intervention effects were found in the full or restricted samples. For child daily %Efat in the full sample, children in the PH-only condition consumed significantly more %Efat than children in the control condition (M = 36.01 vs. M = 34.94, *p* = 0.04). No significant effect was found in the restricted sample.

Mediators. For caregiver dietary intake, caregivers in the HC-only condition reported consuming significantly less %Efat compared to those in the control condition (M = 30.13 vs. M = 31.49; *p* = 0.012) in the full sample at follow-up. There were no significant intervention effects in either the full or restricted samples on caregiver FV intake or any of the parenting strategies. For family meal context mediators, HC-only caregivers reported significantly fewer days of consuming foods prepared at restaurants in the past week compared with control caregivers in the full (M = 1.13 vs. M = 1.46; *p* = 0.007), as well as in the restricted (M = 1.07 vs. M = 1.50; *p* = 0.007) samples. Regarding family meals together, HC-only caregivers reported significantly fewer days of eating family breakfast together in the past week compared with control caregivers in the full (M = 3.14 vs. M = 3.69; *p* = 0.039) and the restricted sample (M = 3.11; vs. M = 3.96; *p* = 0.034). Intervention effects were not observed on other family meal context variables (see Table 3).

### 3.4. Mediating Role of Caregiver Dietary Intake, Parenting Strategies, and Family Meal Contexts

Mediation analysis was performed for only one child dietary outcome that showed positive effects from the intervention (i.e., HC-only children had greater fruit intake compared to those in the control condition among the restricted sample). The significant mediator that showed positive effects from the HC-only intervention was included in the model (i.e., HC-only caregivers reported eating foods from restaurants on fewer days compared to those in the control condition among the restricted sample). The HC-only intervention effect on fruit intake was not mediated by frequency of consumption of foods prepared by restaurants (ab = −0.004; 95% CI: [−0.06, 0.067]).

## 4. Discussion

A significant intervention effect was found for the PH-only condition among the full sample. In contrast to expectation, children in the PH-only condition consumed more %Efat at 12-month follow-up than children in the control condition. This unexpected finding may partly be explained by food acculturation. Caregivers in the PH-only condition were potentially more acculturated to the U.S. diet as indicated by the highest percentage of caregivers who only speak English among all conditions (See Table 2). One study found that the diet of Mexican Americans born in the U.S. contain more fat than the more traditional diet consumed by Mexican Americans born in Mexico [44]. In addition, as indicated earlier in the methods section, a significant portion of children under five were not exposed to PH intervention activities in the ECE centers. While children in the PH-only condition may have been exposed to the interventions in the community recreation organizations and restaurants, their lack of exposure to the intervention through ECE centers could have impacted dietary outcomes. Further, children who were overweight/obese in the HC-only condition consumed significantly more cups of fruit compared to those who were in the control condition at follow-up. No intervention effects were seen for child vegetable intake. These findings are consistent with a meta-analysis of 27 school-based interventions to improve daily FV intake among children aged 5–12 years old [4]. Results showed that FV improvements were mainly due to fruit versus vegetable intake and the gains in fruit intake among children were because of its versatility as breakfast and snacks [45].

The mechanisms of intervention effects were explored through examination of several potential mediators. Consistent with previous obesity prevention studies [17], caregiver %Efat was lower in the HC-only condition compared to the control. In addition, less frequent consumption of foods prepared from restaurants was observed in the HC-only condition; this is a promising finding given that eating out at restaurants is a modifiable risk factor for childhood obesity that can be targeted via strategies such as parent–child cooking classes [46]. In contrast, HC-only families had significantly fewer days of eating family breakfast together compared to the control families. While two previous obesity prevention trials [38,47] targeting Hispanic/Latino children and mothers did not find a significant intervention effect on family meals eaten together, the significant finding was unexpected. Family meal practices may be impacted by factors not assessed in this study, such as parent work schedules.

In general, no significant intervention effects were found in the HC + PH condition, which was hypothesized to produce the most impactful effects due to the synergistic nature of the intervention. This could be partly due to the lack of exposure to the PH strategies discussed earlier. However, caregivers/children in the HC-only condition did show significant effects on fruit intake and other potential mediators. The HC intervention had additional components that reinforced the intervention content such as the four-point treatment plan and family wellness program. It is possible that these attempts to engage caregivers (e.g., negotiating the four-point treatment plan with the provider; participating in the workshops with their children) led to the positive outcomes observed in the HC-only condition. In past studies, the use of behavior change techniques and greater caregiver involvement showed more promising results than studies using strategies that did not require active caregiver engagement [48]. Finally, participants in the HC-only condition may have performed better than those in the HC + PH condition because the HC-only condition was implemented in the city where there was an active clinical champion.

Finally, the effect of HC-only intervention on child fruit intake was not mediated by the frequency of consumption of foods prepared by the restaurant. A similar result was found previously: away-from-home food did not mediate the increase in child FVs intake as a result of the family intervention component of an earlier longitudinal trial [19].

### Study Strengths and Limitations

The present study has several strengths. First, this study evaluates an intervention with opportunities for direct involvement of both children and caregivers. Second, only a few studies to date [19,47] have assessed the mediating role of family meal context in childhood obesity prevention and control interventions. Third, most previous studies assessing the relationship between child and caregiver dietary intake relied on cross-sectional data [14,15], while the present study examines the longitudinal assessment of intervention effect on child/caregiver dietary intake, accounting for their baseline dietary consumption.

There are also several study limitations. First, confounding and selection bias are likely to occur in a design without randomization; however, characteristics that were significantly different across conditions were adjusted in the models to minimize these threats. Second, child dietary intake was subject to proxy reporting and desirability bias. Third, caregiver %Efat (NCI Fat Screener) was not assessed the same way as child %Efat (BKFS) and the timeframe for reporting past consumption of foods and beverages was different: past year versus past week, respectively. Although the NCI Fat Screener is not as comprehensive as the BKFS, it is a validated instrument to assess daily %Efat [49]. Fourth, a high percentage (PH-only 64.7%; HC + PH: 68.4%) of enrolled children under the age of five years were not exposed to the ECE intervention, reducing the planned intervention dose. Finally, this study focused on children living in rural communities close to the U.S.–Mexico border, which may limit generalizability.

## 5. Conclusions

In comparison to the control condition, the HC-only condition showed slightly more fruit intake among the restricted sample (i.e., children who were overweight/obese) and less frequent consumption of foods prepared from the restaurants at the 12-month follow-up in both full and restricted samples. Neither the PH-only nor HC + PH condition showed any positive impact on the dietary intake or the proposed mediators. This and a previous investigation [19] were unable to demonstrate that consumption of away-from-home foods mediates the intervention effect on FV consumption. Finally, Hispanic/Latino children participating together with their caregivers in an obesity prevention and control intervention integrated into a healthcare system utilizing the Obesity Care Model were able to achieve minor behavior modifications such as higher child fruit intake and less frequent family consumption of away-from-home foods.

### Future Directions

This study makes many important contributions to the literature by describing the implementation and impact of a largescale, multisector, multilevel intervention conducted in a rural, border region with predominantly Hispanic/Latino families—a community that is often underrepresented in public health nutrition research. While some dietary improvements were observed among participants in this study, continued efforts are needed to identify best practices for improving diet quality within this community. In particular, future researchers can expand upon this work by considering the following methodological changes: (a) utilize participant randomization to avoid confounding and selection biases; (b) use comparable child and caregiver dietary intake data to ensure equivalence of the measures; (c) include a greater depth of family meal context variables for analysis (e.g., mealtime routines, caregiver/child mealtime media use, family mealtime duration). Further, additional intervention strategies (e.g., media campaigns or educational materials) could be developed to focus on aspects of diet that did not significantly improve in this study, such as vegetable and fat intake. Finally, researchers could adapt and evaluate the impact of similar intervention strategies in other communities and with older children.

## Figures and Tables

**Table 1:** Diet-related intervention strategies used in the HC condition (HC + PH and HC-only) and PH conditions (HC + PH and PH-only) within a multisector and multilevel obesity intervention study (CA-CORD).

	Targeted Sector	Intervention Activities Related to Dietary Intake		
		Capacity Building	Policy, System, and Environmental Changes	Media Campaign
Healthcare Condition	Federally Qualified Health Center (FQHC) (N = 1; 3 clinic sites)	Trained healthcare providers and a hired and trained patient care coordinator implemented the Obesity Care Model (OCM). Hired and trained 7 CHWs and a CHW coordinator to conduct the family wellness program (FWP): behavior change workshops, physical activity workshops, and motivational interview (MI) calls.	Approved four new policies for childhood obesity prevention and control. Providers and patient care coordinatorhelped families set goals for four health behaviors *. Providers entered health behavior goals into the EHR system to provide the families with a four-point treatment plan. Providers referred children and caregivers to the FWP. CHWs delivered the FWP: ○ Six group-based behavior change workshops (e.g., add more fruits and vegetables; substitute unhealthy snacks (high-fat, high-energy-dense foods) for healthy ones (low-fat, low-energy-dense foods); modify restaurant choices; eat meals as family, etc.). Followed by 20–30 min of physical activity. ○ Four MI calls. ○ Twelve newsletters; each was sent monthly	Provided posters promoting health behaviors * to each clinic site.
Public Health Condition	ECE Centers (N = 23)	Provided health behavior training and a health behavior toolkit to help staff promote the four health behaviors *. Provided incentives to centers whose staff attended the training sessions.	Conducted Nutrition and Physical Activity Self-Assessment for Child Care (NAP SACC) and action planning at each center to improve food policies and the food environment. Provided a child-friendly cooking kit to promote child engagement in cooking and serving meals. Provided a water dispenser to increase water access for children.	Provided posters promoting health behaviors * to each ECE center.
	Elementary Schools (N = 13)	Presented monthly health behavior themes at teacher/staff meetings. Provided monthly tip sheets for teachers to integrate into healthy lifestyle messages for students.	Implemented school wellness policy changes (e.g., replace unhealthy foods and beverages with healthy alternatives at classroom parties; rewards with healthy options for holiday celebrations). Provided water bottles and water drinking handouts to distribute to students. Provided water container(s) or water jets (in one school district) to increase water access for students.	Encouraged schools to implement water promotion campaigns. Provided posters promoting health behaviors * to each school.
	Community Recreation Organizations (N = 3)	Provided two 4 h training sessions and technical assistance to recreation site staff to promote the health behaviors *, work toward policy changes in needed areas, and make environmental changes. Provided a workshop to staff and community members who help maintain the community garden in two recreation organizations.	Developed a community garden in two organizations and provided garden supplies. Provided a water dispenser to each organization to increase water access for children.	Provided posters promoting health behaviors * to each organization.
	Restaurants (N = 3)	Trained kitchen staff and wait staff on the preparation and promotion of new healthy child menu.	Modified existing child menus (n = 2) or introduced a new healthy child menu (n = 1) to increase access to healthier foods and beverages for children.	Provided a marketing campaign (e.g., new menu, posters, etc.) to promote the new children’s menu at each restaurant.

FQHC = Federally Qualified Health Center; OCM = Obesity Care Model; FWP = family wellness program; ECE = Early Care and Education. * Health behaviors refer to four targeted health behaviors in the CA-CORD study: dietary intake (including reducing fat intake and increasing fruit and vegetable intake), physical activity, water intake, and sleep.

**Table 2:** Baseline characteristics of 1186 children and their caregivers (N = 848) and families (N = 848) across four conditions of a multisector and multilevel obesity intervention study (CA-CORD): control, healthcare only (HC-only), public health only (PH-only), and HC + PH.

	Overall (N = 1186) ^a^	Control (N = 272)	HC-Only (N = 276)	PH-Only (N = 308)	HC + PH (N = 330)	*p*-Value
	Mean (SD) or % (n)	Mean (SD) or % (n)	Mean (SD) or % (n)	Mean (SD) or % (n)	Mean (SD) or % (n)	
Child characteristics						
Female	50.5% (599)	56.3% (153)	52.2% (144)	49.4% (152)	45.5% (150)	0.06
Age	6.4 (2.7)	6.7 (2.7)	6.5 (2.7)	6.0 (2.6)	6.5 (2.7)	<0.05
Latino/Hispanic ^b^	98.5% (1165)	99.3% (270)	99.3% (274)	97.7% (300)	97.9% (321)	0.23
Foreign-born ^b^	8.3% (98)	7.0% (19)	13.8% (38)	4.2% (13)	8.5% (28)	<0.01
Only spoke English ^b^	9.4% (111)	3.3% (9)	2.2% (6)	19.5% (60)	10.9% (36)	<0.01
Daily cups fruit intake ^b^	1.4 (0.9)	1.3 (0.8)	1.4 (0.9)	1.4 (0.9)	1.4 (0.9)	0.76
Daily cups vegetable intake ^b^	0.6 (0.4)	0.6 (0.4)	0.6 (0.4)	0.6 (0.4)	0.6 (0.4)	0.49
Daily percentage energy from fat (%Efat) ^b^	35.0 (4.6)	35.0 (4.2)	35.0 (4.9)	35.2 (4.6)	34.7 (4.7)	0.54
BMI percentile	76.3 (26.5)	72.9 (27.9)	78.4 (26.1)	75.8 (26.4)	77.9 (25.5)	0.05
Parenting strategies ^c^						
Monitoring	4.1 (0.9)	4.2 (0.8)	4.0 (0.9)	4.1 (0.9)	4.1 (0.9)	0.09
Limit setting	4.4 (1.0)	4.4 (0.9)	4.3 (1.0)	4.4 (0.9)	4.5 (1.0)	0.23
Discipline ^b^	2.7 (1.3)	2.8 (1.4)	2.6 (1.3)	2.8 (1.4)	2.7 (1.3)	0.47
Control	3.1 (1.0)	3.1 (1.0)	3.1 (1.0)	3.1 (1.0)	3.1 (1.1)	0.78
Reinforcement ^b^	3.3 (1.5)	3.4 (1.5)	3.0 (1.5)	3.5 (1.4)	3.2 (1.5)	<0.01
Permission seeking	3.8 (1.2)	3.8 (1.2)	3.7 (1.2)	4.0 (1.1)	3.8 (1.4)	<0.01
	Overall (N = 848) ^a^	Control (N = 195)	HC-only (N = 201)	PH-only (N = 223)	HC + PH (N = 229)	*p*-value
Caregiver characteristics						
Female	98.4% (834)	98.0% (191)	98.5% (198)	98.2% (219)	98.7% (226)	0.94
Relationship to child						0.47
Mother	93.2% (790)	92.8% (181)	93.0% (187)	91.5% (204)	95.2% (218)	
Other relatives	6.8% (58)	7.2% (14)	7.0% (14)	8.5% (19)	4.8% (11)	
Age ^d^	35.7 (8.5)	37.0 (9.1)	36.1 (8.8)	34.8 (8.3)	35.0 (7.6)	<0.05
Latino/Hispanic ^d^	97.5% (824)	98.0% (191)	99.0% (198)	95.5% (211)	97.8% (224)	0.12
Foreign-born ^c^	67.1% (568)	66.7% (130)	75.1% (151)	57.5% (127)	69.9% (160)	<0.01
Only spoke English ^d^	9.1% (77)	5.1% (10)	2.5% (5)	19.4% (43)	8.3% (19)	<0.01
Married ^d^	72.7% (613)	77.8% (151)	69.2% (139)	74.2% (164)	70.0% (159)	0.18
Education ^d^						<0.01
Less than high school	31.7% (268)	25.6% (50)	35.8% (72)	24.1% (53)	40.6% (93)	
High school and above	68.3% (577)	74.4% (145)	64.2% (129)	75.9% (167)	59.4% (136)	
Employed ^d^	39.5% (334)	45.1% (88)	39.8% (80)	44.3% (98)	29.7% (68)	<0.01
Owns a home ^d^	21.9% (185)	30.9% (60)	16.4% (33)	26.2% (58)	14.9% (34)	<0.01
Daily cups fruit intake ^d^	1.6 (1.3)	1.7 (1.3)	1.7 (1.3)	1.6 (1.3)	1.6 (1.4)	0.59
Daily cups vegetable intake ^d^	2.0 (1.3)	2.0 (1.4)	2.1 (1.3)	2.0 (1.3)	1.9 (1.3)	0.69
Daily percentage energy from fat (%Efat) ^d^	31.2 (4.2)	31.2 (3.9)	31.1 (4.5)	31.1 (4.1)	31.2 (4.4)	0.99
BMI (kg/m^2^) ^d^	31.8 (7.2)	30.3 (6.2)	31.4 (7.0)	32.6 (7.5)	32.6 (7.6)	<0.01
Family characteristics						
Household size ^e^	4.7 (1.5)	4.7 (1.3)	4.8 (1.5)	4.7 (1.4)	4.8 (1.6)	0.37
Ratio of family income to poverty (PIR) ^e,f^	0.9 (0.8)	1.2 (1.0)	0.7 (0.5)	1.1 (0.9)	0.8 (0.6)	<0.01
Food assistance ^e,g^	70.6% (597)	58.0% (113)	74.1% (149)	63.4% (140)	85.2% (195)	<0.01
Family diet-context						
Days past week food from relatives’ or friends’ home	1.0 (1.3)	0.9 (1.2)	1.1 (1.4)	1.0 (1.3)	0.9 (1.3)	0.34
Days past week food from restaurants	1.3 (1.1)	1.3 (1.0)	1.3 (1.1)	1.4 (1.1)	1.2 (1.1)	0.37
Days past week family morning meal ^e^	3.7 (2.5)	3.4 (2.4)	3.9 (2.6)	3.6 (2.5)	3.7 (2.6)	0.08
Days past week family afternoon meal/snack ^e^	5.3 (2.2)	5.0 (2.4)	5.7 (1.8)	5.1 (2.4)	5.5 (2.2)	<0.01
Days past week family evening meal ^e^	5.3 (2.3)	4.9 (2.5)	5.4 (2.2)	5.5 (2.1)	5.4 (2.2)	<0.01

^a^ Up to two children per family can be enrolled into the study; thus, the total number of children and caregivers/family is different. ^b^ Missing data for children were observed for the following variables: Latino ethnicity 0.3%; foreign-born 0.1%; only spoke English 0.1%; fruit intake 0.1%; vegetable intake 0.1%; %Efat 0.1%; PEAS: discipline 0.7%; PEAS: reinforcement 0.4%. ^c^ Parenting strategies for eating and activity scale (PEAS); response was provided for each individual child in this study. The response options vary depending on the strategies: 1 (never)–5 (always) for monitoring, discipline, reinforcement, and permission-seeking strategies; 1 (disagree)–5 (agree) for limit setting and control strategies. ^d^ Missing data for caregivers were observed for the following variables: age 0.2%; Latino ethnicity 0.4%; foreign-born 0.2%; only spoke English 0.1%; marital status 0.6%; education 0.4%; employment status 0.2%; home ownership 0.4%; fruit intake 0.2%; vegetable intake 0.2%; %Efat 1.3%; BMI 0.1%. ^e^ Missing data for families were observed for the following variables: household size 0.2%; PIR 5.3%; participation in food assistance program 0.2%; number of days a week the family ate a morning meal together 0.3%; number of days a week a family ate an afternoon meal/snack together 0.4%; number of days a week a family ate an evening meal together 0.2%. ^f^ The United States (U.S.) Department of Health and Human Services’ 2011 poverty guidelines were used to obtain poverty cutoff values to calculate the PIR. A ratio of 1.0 or greater indicated the income was above the poverty level and a ratio lower than 1.0 indicated the opposite. ^g^ Participation in the food assistance program such as SNAP or WIC.

**Table 3:** The CA-CORD intervention effects on children’s daily diet (cups of fruit, cups of vegetable, percent energy from fat) and potential intervention mediators such as caregiver dietary intake, parenting strategies, and family meal context for both full and restricted samples: Adjusted mean at 12-month follow-up.

	Full Sample (All Children)						Restricted Sample (Children with BMI Percentile ≥ 85%)					
	n	Adjusted Mean	β	SE	*p*-Value	Adjusted *p*-Value ^a^	n	Adjusted Mean	β	SE	*p*-Value	Adjusted *p*-Value ^a^
CHILD DIET OUTCOMES												
Child daily cups fruit	931						513					
Control	227	1.16	-	-	-	-	108	1.09	-	-	-	-
HC-only	206	1.31	0.15	0.08	0.051	0.051	123	1.32	0.23	0.10	0.020	0.040
PH-only	241	1.17	0.01	0.07	0.918	0.918	130	1.06	−0.02	0.10	0.809	0.918
HC + PH	257	1.15	−0.01	0.07	0.891	0.891	152	1.15	0.06	0.10	0.507	0.891
Child daily cups vegetables	931						513					
Control	227	0.57	-	-	-	-	108	0.55	-	-	-	-
HC-only	206	0.56	−0.01	0.04	0.785	0.968	123	0.55	0.00	0.04	0.968	0.968
PH-only	241	0.57	0.00	0.03	0.993	0.993	130	0.53	−0.03	0.04	0.503	0.993
HC + PH	257	0.56	−0.01	0.04	0.737	0.854	152	0.56	0.01	0.04	0.854	0.854
Child daily percentage energy from fat (%Efat)	931						513					
Control	227	34.94	-	-	-	-	108	35.51	-	-	-	-
HC-only	206	34.49	−0.45	0.48	0.349	0.349	123	34.11	−1.40	0.64	0.028	0.056
PH-only	241	36.01	1.07	0.46	0.020	0.040	130	36.20	0.69	0.63	0.274	0.274
HC + PH	257	35.40	0.45	0.46	0.329	0.658	152	35.62	0.11	0.62	0.861	0.861
MEDIATORS: CAREGIVER DIETARY INTAKE												
Caregiver daily cups fruit	669						380					
Control	161	1.68	-	-	-	-	82	1.52	-	-	-	-
HC-only	152	1.83	0.16	0.15	0.289	0.289	96	1.90	0.38	0.19	0.043	0.086
PH-only	175	1.55	−0.13	0.14	0.362	0.724	94	1.47	−0.04	0.19	0.818	0.818
HC + PH	181	1.59	−0.08	0.14	0.564	0.953	108	1.53	0.01	0.19	0.953	0.953
Caregiver daily cups vegetable	669						380					
Control	161	1.97	-	-	-	-	82	1.75	-	-	-	-
HC-only	152	2.01	0.04	0.14	0.773	0.773	96	1.92	0.17	0.19	0.364	0.728
PH-only	175	1.80	−0.17	0.14	0.221	0.442	94	1.68	−0.07	0.19	0.721	0.721
HC + PH	181	1.93	−0.04	0.14	0.785	0.785	108	1.97	0.22	0.19	0.236	0.472
Caregiver daily percentage energy from fat (%Efat)	655						373					
Control	159	31.49	-	-	-	-	82	30.83	-	-	-	-
HC-only	148	30.13	−1.36	0.50	0.006	0.012	92	30.46	−0.37	0.66	0.576	0.576
PH-only	170	31.42	−0.07	0.47	0.886	0.886	93	31.73	0.91	0.65	0.161	0.322
HC + PH	178	30.97	−0.52	0.48	0.278	0.556	106	31.04	0.21	0.65	0.738	0.738
MEDIATORS: PARENTING STRATEGIES ^b^												
PEAS: Monitoring	931						512					
Control	227	4.07	-	-	-	-	108	4.14	-	-	-	-
HC-only	205	4.17	0.10	0.09	0.254	0.443	122	4.22	0.09	0.11	0.443	0.443
PH-only	241	4.10	0.03	0.09	0.759	0.813	130	4.11	−0.03	0.11	0.813	0.813
HC + PH	258	4.21	0.14	0.09	0.107	0.214	152	4.23	0.09	0.11	0.400	0.400
PEAS: Limit setting	932						513					
Control	227	4.44	-	-	-	-	108	4.44	-	-	-	-
HC-only	206	4.60	0.15	0.10	0.114	0.114	123	4.67	0.23	0.12	0.047	0.094
PH-only	241	4.44	0.00	0.09	0.997	0.997	130	4.55	0.12	0.11	0.317	0.634
HC + PH	258	4.49	0.05	0.09	0.572	0.572	152	4.55	0.11	0.11	0.331	0.572
PEAS: Discipline	923						508					
Control	224	2.50	-	-	-	-	107	2.69	-	-	-	-
HC-only	205	2.43	−0.07	0.14	0.612	0.612	122	2.54	−0.14	0.18	0.419	0.612
PH-only	239	2.73	0.24	0.13	0.072	0.144	129	2.89	0.21	0.17	0.230	0.230
HC + PH	255	2.70	0.20	0.13	0.127	0.254	150	2.75	0.06	0.17	0.719	0.719
PEAS: Control	932						533					
Control	227	2.92	-	-	-	-	108	2.72	-	-	-	-
HC-only	206	2.88	−0.04	0.10	0.683	0.692	123	2.67	−0.05	0.12	0.692	0.692
PH-only	241	2.82	−0.10	0.09	0.289	0.289	130	2.54	−0.18	0.12	0.128	0.256
HC + PH	258	2.81	−0.11	0.09	0.249	0.283	152	2.60	−0.12	0.12	0.283	0.283
PEAS: Reinforcement	927						510					
Control	224	2.99	-	-	-	-	106	3.02	-	-	-	-
HC-only	205	3.30	0.32	0.15	0.041	0.063	122	3.38	0.37	0.20	0.063	0.063
PH-only	241	3.26	0.27	0.15	0.066	0.132	130	3.08	0.06	0.19	0.756	0.756
HC + PH	257	3.23	0.25	0.15	0.100	0.200	152	3.25	0.23	0.19	0.227	0.227
PEAS: Permission seeking	931						512					
Control	227	3.61	-	-	-	-	108	3.62	-	-	-	-
HC-only	205	3.76	0.15	0.13	0.248	0.456	122	3.74	0.13	0.17	0.456	0.456
PH-only	241	3.68	0.07	0.13	0.557	0.557	130	3.73	0.12	0.17	0.484	0.557
HC + PH	258	3.78	0.17	0.13	0.192	0.384	152	3.76	0.14	0.16	0.388	0.388
MEDIATORS: FAMILY MEAL CONTEXT												
Days past week food from relatives’ or friends’ home	668						379					
Control	160	1.21	-	-	-	-	81	1.18	-	-	-	-
HC-only	152	0.99	−0.22	0.16	0.180	0.360	96	1.01	−0.17	0.21	0.418	0.418
PH-only	175	1.27	0.06	0.15	0.699	0.717	94	1.25	0.08	0.21	0.717	0.717
HC + PH	181	1.05	−0.16	0.16	0.296	0.592	108	1.17	−0.01	0.21	0.950	0.950
Days past week food from restaurants	667						378					
Control	159	1.46	-	-	-	-	80	1.50	-	-	-	-
HC-only	152	1.13	−0.33	0.12	0.005	0.007	96	1.07	−0.43	0.16	0.007	0.007
PH-only	175	1.34	−0.12	0.11	0.276	0.276	94	1.23	−0.28	0.16	0.081	0.162
HC + PH	181	1.36	−0.10	0.11	0.372	0.407	108	1.37	−0.13	0.16	0.407	0.407
Days past week family morning meal	668						379					
Control	161	3.69	-	-	-	-	82	3.96	-	-	-	-
HC-only	152	3.14	−0.55	0.27	0.039	0.039	96	3.11	−0.85	0.35	0.017	0.034
PH-only	174	4.20	0.51	0.25	0.046	0.092	93	4.38	0.42	0.35	0.227	0.227
HC + PH	181	3.46	−0.24	0.26	0.361	0.361	108	3.57	−0.39	0.35	0.266	0.361
Days past week family afternoon meal/snack	667						379					
Control	160	5.19	-	-	-	-	81	5.27	-	-	-	-
HC-only	152	4.92	−0.27	0.25	0.272	0.544	96	5.08	−0.19	0.34	0.583	0.583
PH-only	175	5.37	0.19	0.23	0.427	0.854	94	5.21	−0.06	0.33	0.867	0.867
HC + PH	180	5.19	0.00	0.24	0.996	0.996	108	5.24	−0.03	0.33	0.937	0.996
Days past week family evening meal	669						380					
Control	161	5.42	-	-	-	-	82	5.17	-	-	-	-
HC-only	152	5.24	−0.18	0.23	0.439	0.878	96	5.21	0.04	0.32	0.904	0.904
PH-only	175	5.28	−0.14	0.22	0.517	0.920	94	5.14	−0.03	0.32	0.920	0.920
HC + PH	181	5.25	−0.17	0.23	0.444	0.888	108	5.18	0.01	0.31	0.966	0.966

^a^ The *p*-value was adjusted with Benjamini–Hochberg method for each family of the outcome/mediator. The significance of the intervention effect was determined based on this *p*-value. ^b^ Parenting strategies for eating and activity scale (PEAS); response was provided for each individual child in this study. The response options vary depending on the strategies: 1 (never)–5 (always) for monitoring, discipline, reinforcement, and permission-seeking strategies; 1 (disagree)–5 (agree) for limit setting and control strategies.

## Data Availability

The data presented in this study are not available to share. Analyses are ongoing and findings will be presented in forthcoming manuscripts.
